# ADAMTS13 activity decreases in the early phase of trauma associated with coagulopathy and systemic inflammation: a prospective observational study

**DOI:** 10.1186/s12959-021-00270-1

**Published:** 2021-03-12

**Authors:** Hironori Matsumoto, Jun Takeba, Kensuke Umakoshi, Satoshi Kikuchi, Muneaki Ohshita, Suguru Annen, Naoki Moriyama, Yuki Nakabayashi, Norio Sato, Mayuki Aibiki

**Affiliations:** Department of Emergency and Critical Care Medicine, Ehime University, Graduate School of Medicine, Shitsukawa 454, Toon City, Ehime 791-0295 Japan

**Keywords:** Albumin, Antithrombin, Coagulofibrinolysis, Disseminated intravascular coagulation (DIC), Extravascular leakage

## Abstract

**Background:**

We conducted a prospective observational study for investigating the changes in the 13th member of a disintegrin-like and metalloprotease with thrombospondin type 1 motif (ADAMTS13) and its association with the coagulofibrinolytic response in adult trauma patients.

**Methods:**

In 39 trauma patients hospitalized for longer than 7 days, time-course changes in biomarkers of coagulofibrinolysis and systemic inflammation along with ADAMTS13 activity were examined. The patients were stratified into three groups based on ADAMTS13 activities on admission (day 0): normal group (≥70%), mildly decreased group (≥50 and < 70%) and moderately decreased group (< 50%).

**Results:**

Among 39 patients with a median Injury Severity Score (ISS) of 20, 11 patients developed disseminated intravascular coagulation (DIC) and 16 patients required transfusion. Six of 39 patients (15.4%) showed moderate decreased ADAMTS13 activity to < 50%, and 20 patients (51.3%) showed mild drops (≥50 and < 70%). These changes in ADAMTS13 activity on day 0 were significantly correlated with changes in IL-6 and other coagulofibrinolytic markers such as platelet counts, prothrombin time and fibrin/fibrinogen degradation product (FDP). Antithrombin activity (AT) and serum albumin (Alb) level showed significantly positive linear correlations with ADAMTS13 activity (AT: *r* = 0.513, *p* < 0.001; Alb: *r* = 0.647, *p* < 0.001). Simple logistic regression analyses showed that ADAMTS13 activity, if less than 50%, was significantly correlated with the development of DIC (*OR* 7.499, 95%*CI* 1.121–49.242, *p* = 0.038) and the need for transfusion of fresh frozen plasma (*OR* 9.000, 95%*CI* 1.327–61.025, *p* = 0.028).

**Conclusions:**

ADAMTS13 activity decreased even in the early phase of trauma, which was complicated by coagulopathy and systemic inflammation. Furthermore, the decrease in ADAMTS13 activity was correlated with DIC and plasma transfusion.

## Background

The 13th member of a disintegrin-like and metalloprotease with thrombospondin type 1 motif (ADAMTS13) is secreted from hepatic stellate and endothelial cells, and reduces von Willebrand factor (vWF) coagulation activities by cleaving vWF multimers into small fragments, and thus plays an important role in regulating thrombosis. vWF is mainly synthesized from endothelial cells and megakaryocytes, and is then stored in Weibel-Palada bodies and platelet α granules. The bioactivity of vWF’s coagulation function depends on its molecular weight; its higher molecular type exhibits more powerful activities. The monomer of vWF is 250 kDa, and vWFs present in plasma with molecular weight of 500-15,000 kDa. vWF multimers in the storage granules of endothelial cells are rich in a highly thrombogenic form of unusually large vWF multimers (ULvWF). If endothelial cells were injured, ULvWF will be released from the granules into the blood, and facilitates platelet adhesion and aggregation, then cleaved by ADAMTS13 as stated above. Therefore, deficiency of ADAMTS13 could cause the formation of microvascular thrombosis [1–6]. Thrombotic microangiopathy (TMA) is a pathological diagnosis established by the presence of thrombosis in small vessels and involves thrombotic thrombocytopenic purpura (TTP) and hemolytic uremic syndrome (HUS) [4, 7]. Severe ADAMTS13 deficiency such as hereditary or acquired TTP provokes life-threatening thrombotic sequelae. On the other hand, some acute critical diseases such as sepsis are often complicated by a secondary mild to moderate decrease in ADAMTS13 levels, which is thought to be one of the heterogenic reactions, even without the development of TMA or TTP [4, 8]. Secondary ADAMTS13 deficiency is considered to develop as a result of endothelial cell injury [9], and also this deficiency is known to be well associated with thrombotic disorders and severity or prognosis of primary diseases. Regarding trauma, in a pediatric study, ADAMTS13 decreased with vWF elevation, which was associated with coagulopathy and endothelial cell injury [10]. Nevertheless, the changes in ADAMTS13 levels in a trauma setting have not been sufficiently elucidated. The aim of this study is to examine the changes in ADAMTS13 activity and to evaluate its association with coagulofibrinolysis and inflammation in adult trauma patients.

## Methods

### Study design

We performed a prospective observational study by collecting the data of adult trauma patients admitted to the tertiary Ehime University Hospital in Japan from January 2015 to April 2016. This study was approved by the Institutional Local Ethics Committee for Clinical Studies. Informed consent was obtained from all patients or next of kin in accordance with the Declaration of Helsinki.

### Patient selection and criteria

All adult trauma patients (≥18 years) who were immediately admitted to our hospital, or were transferred from other hospitals without any significant therapeutic interventions, and were estimated to require hospitalization for more than 7 days were enrolled. We excluded patients who had already received therapeutic interventions including infusion (more than 500 mL of fluid administration or medications before transfer to our hospital), those who had died during initial treatments at the emergency department, those who had an episode of cardiac arrest, those who received anticoagulant therapy, and those who had a clotting disorder such as liver cirrhosis or advanced malignancies.

Demographic data, examinations, treatments, and mortality were recorded. Trauma usually causes dynamic coagulofibrinolytic changes. Excessive thrombin generation and remarkably enhanced fibrinolysis beyond the physiological response lead to a pathophysiological condition such as trauma-induced coagulopathy (TIC) and disseminated intravascular coagulation (DIC) [11]. The pathological coagulopathy would be clearly distinguished from the physiological hemostasis especially when DIC diagnosis criteria were used [12]. We therefore used DIC criteria for the assessment of the patients who developed pathological coagulopathy. Systemic inflammatory response syndrome (SIRS) was defined according to the consensus conference of the American College of Chest Physicians/Society of Critical Care Medicine [13]. The diagnosis for DIC was made based on the Japanese Association for Acute Medicine (JAAM) DIC criteria [14], according to which, patients with a score of at least 4 were diagnosed with DIC. Deep venous thrombosis was diagnosed by ultrasonography on day 6. The decision and timing for initiating pharmacological VTE prophylaxis were at the discretion of the physicians in charge after the evaluation of the patients’ condition. Transfusions of packed red blood cells, fresh-frozen plasma, or platelets were allowed in order to maintain the hemodynamics and the hemostasis at the discretion of the clinicians informed by the laboratory data.

The normal ADAMTS13 activity is 100%, ranging from 70 to 120%. Thus, we stratified the patients into three groups according to the ADAMTS13 activities on arrival (day 0): normal (≥70%); slightly decreased, showing less than the lower normal limit (≥50 and < 70%); and moderately decreased, showing less than half the normal activity (< 50%).

### Blood sampling and measurement

Blood sampling was performed immediately upon arrival (day 0) and on days 1, 2, 4, and 6. We routinely measured blood counts and biochemistries including albumin (Alb), and C-reactive protein (CRP) with TBA-c16000 (Toshiba Medical Systems, Tochigi, Japan) and XE-5000 (Sysmex, Hyogo, Japan). We also measured the biomarkers of coagulofibrinolysis using CP-2000 (Sekisui Medical, Tokyo, Japan) and STACIA (LSI Medience, Tokyo, Japan): prothrombin time (PT), activated partial thromboplastin time (APTT), hepaplastin test (HPT), fibrinogen (Fbg), fibrin/fibrinogen degradation product (FDP), D-dimer, soluble fibrin (SF), thrombin-antithrombin complex (TAT), plasmin-α_2_-plasmin inhibitor complex (PIC), antithrombin (AT), protein C (PC), α_2_-plasmin inhibitor (α_2_PI), and plasminogen (PLG). After sampling, the blood samples were centrifuged at 3300 rpm for 15 min at 4 °C, and serum and plasma samples were stored at − 80 °C for subsequent analyses. We measured total plasminogen activator inhibitor-1 (tPAI-1) on days 0, 1, 2 and 6; soluble thrombomodulin (sTM) and ADAMTS13 activity on days 0, 2 and 6; and IL-6 on day 0. tPAI-1 represents the sum of active PAI-1, t-PA/PAI-1 and latent PAI-1, and was measured by the LPIA·tPAI-1 test with the automated immunoanalyzer STACIA (LSI Medience Corporation, Tokyo, Japan). sTM was measured by one-step sandwich enzyme immunoassay using a monoclonal antibody specific for thrombomodulin (TM Panacella; Fujirebio, Tokyo, Japan). ADAMTS13 activity was measured by enzyme immunoassay (ADAMTS13 Activity ELISA Kit; KAINOS Laboratories, Inc., Tokyo, Japan) [15]. IL-6 was measured by enzyme-linked immunosorbent assay (Human IL-6 Quantikine ELISA kit, R&D Systems, Minneapolis, MN, USA).

### Statistical analysis

Statistical analyses were performed using IBM SPSS statistics 22 (IBM, Tokyo, Japan). All data are expressed as median (interquartile range: IQR). A comparison between two of the groups was performed using the Mann-Whitney U test, and a comparison among all three groups was performed using the Kruskal-Wallis test. Time course changes of ADAMTS13 activity during the study period were tested by one-way repeated measures analysis of variance (ANOVA). The longitudinal differences in ADAMTS13 activities during the study period between the subgroups stratified with the activities on day 0 were analyzed by two-way repeated measures ANOVA. The relationships between the dependent variables were analyzed with a linear regression analysis. A stratified logistic regression analysis was used to generate odds ratios (*OR*s) and confidence intervals (*CI*s) for categorical valuables. A *p* value less than 0.05 was considered significant.

## Results

### Characteristics of patients enrolled (Table 1)

Fifty-nine trauma patients were admitted to our hospital during the study period. Twenty patients were excluded based on the exclusion criteria, as stated in the Methods section. As a result, this study included 39 adult trauma patients with a median Injury Severity Score (ISS) of 20 (10–27). The baseline characteristics and coagulofibrinolytic parameters on day 0 are presented in Table 1. The traumatic mechanism for all the patients was blunt injury, so most of them suffered several organ injuries. During the study period, 17 of the patients (43.5%) underwent transfusion, 16 of who (94.1%) received a transfusion on day 0. Therapies such as interventional radiology (IVR), craniotomy, laparotomy, or open reduction and internal fixation (ORIF) were performed on 21 patients (53.8%), ten of who (47.6%) underwent these interventions on day 0. Twenty patients (51.3%) received pharmacological VTE prophylaxis during the course. The development of DVT without related symptoms was observed in six patients (15.3%).

**Table 1:** Patients clinical features and outcomes

		ALL (*n* = 39)	ADAMTS13 activity on day 0			
			50% > (*n* = 6)	50%≤, 70% > (*n* = 20)	70% ≤ (*n* = 13)	*P* value
Patient characteristics						
Age	years	61 (38–73)	71 (67–72)	65 (45–77)	35 (23–56)	0.017
Sex; male / female	n, (%)	26 (66.7) / 13 (33.3)	4 (66.7) / 2 (33.3)	13 (65.0) / 7 (35.0)	9 (69.2) / 4 (30.8)	0.969
Injury Severity Score		20 (10–27)	17 (13–27)	20 (11–28)	20 (10–26)	0.760
JAAM DIC (+)	n, (%)	11 (28.4)	4 (66.7)	5 (25.0)	2 (15.4)	0.063
Duration of injury to blood sampling						
	minutes	68 (31–180)	43 (31–136)	121 (44–261)	41 (28–92)	0.134
Laboratory data [normal range]						
HGB [11.3–15.2]	g/dL	12.8 (11.8–13.6)	12.0 (10.3–12.2)	12.0 (10.9–12.9)	13.6 (13.3–14.8)	< 0.001
PLT [13.1–36.9]	×10^4^/μL	21.6 (16.8–25.3)	12.6 (9.3–18.1)	21.2 (16.7–24.0)	25.3 (21.2–26.6)	0.015
PT [80.0–120.0]	%	84.1 (77.6–97.7)	72.6 (58.8–80.2)	84.1 (80.4–96.6)	90.8 (82.5–114.7)	0.028
APTT [21.5–43.1]	sec	24.7 (23.2–27.1)	26.6 (24.9–30.2)	24.9 (23.5–27.3)	23.3 (22.2–26.5)	0.101
HPT [70.0–130.0]	%	112.7 (91.6–126.8)	85.2 (70.8–91.4)	110.4 (98.9–125.5)	126.4 (101.9–139.9)	0.003
Fbg [200–400]	mg/dL	238 (187–280)	214(181–272)	230 (185–261)	257 (235–304)	0.450
FDP [< 5.0]	μg/mL	63.0 (31.4–168.1)	198.3 (132.2–247.5)	78.3 (31.9–210.8)	46.5 (15.5–59.5)	0.003
D-dimer [< 1.0]	μg/mL	36.4 (16.0–96.6)	107.5 (74.4–136.2)	40.0 (16.3–101.3)	17.7 (7.7–28.2)	0.002
TAT [< 3.0]	μg/L	88.0 (30.1–200.0)	198.5 (114.8–243.1)	106.9 (56.6–200.0)	60.8 (9.8–85.9)	0.056
PIC [0.0–0.8]	μg/mL	9.1 (2.8–17.8)	18.2 (12.6–23.9)	9.5 (3.1–18.0)	4.9 (1.4–9.1)	0.040
tPAI-1 [< 50]	ng/mL	30 (3–602)	89 (40–123)	33 (19–80)	30 (25–48)	0.310
AT [80.0–120.0]	%	96.2 (79.8–108.3)	68.9 (63.8–85.2)	92.2 (83.1–106.5)	103.3 (97.1–110.0)	0.023
PC [82.0–112.0]	%	90.6 (75.8–101.5)	66.0 (47.1–75.4)	90.8 (78.0–102.8)	97.1 (85.0–117.1)	0.021
α_2_PI [80.0–130.0]	%	90.5 (79.6–101.6)	80.4 (72.9–95.9)	84.6 (78.7–92.5)	102.2 (95.6–107.2)	0.003
PLG [80.0–130.0]	%	93.8 (81.5–102.6)	84.7 (70.6–85.6)	92.5 (80.9–100.6)	101.5 (93.8–107.1)	0.071
sTM [1.8–4.1]	F/U	2.9 (2.3–3.7)	3.6 (2.7–3.6)	3.1 (2.5–3.9)	2.4 (2.3–3.0)	0.099
IL-6* [< 4.0]	pg/mL	108.5 (40.8–250.3)	251.0 (191.8–326.8)	119.0 (48.3–216.8)	93.0 (27.8–98.8)	0.037
Lactate [3.3–14.9]	mg/dL	15.3 (10.8–21.6)	14.5 (9.3–21.3)	15.0 (11.0–17.0)	17.0 (13.0–26.0)	0.430
Alb [3.9–4.9]	g/dL	3.9 (3.4–4.3)	3.1 (2.9–3.3)	3.8 (3.4–4.1)	4.3 (4.2–4.6)	< 0.001
ADAMTS13 activity [70.0–120.0]	%	63.6 (56.4–74.5)	45.6 (42.1–47.7)	61.2 (56.5–63.9)	76.7 (75.5–82.5)	< 0.001
Transfusion Day 0 / Day 0–6						
PRBC	Units	0 (0–0) / 0 (0–2)	0 (0–0) / 3 (2–6)	0 (0–1) / 0 (0–4)	0 (0–0) / 0 (0–0)	0.990 / 0.104
FFP	Units	0 (0–2) / 0 (0–2)	2 (0–6) / 2 (0–10)	0 (0–0) / 0 (0–0)	0 (0–0) / 0 (0–0)	0.430 / 0.412
Platelets	Units	0 (0–0) / 0 (0–0)	0 (0–0) / 0 (0–15)	0 (0–0) / 0 (0–0)	0 (0–0) / 0 (0–0)	0.814 / 0.438
Intervention Day 0 / Day 0–6						
IVR	n, (%)	6 (15.3) / 6 (15.3)	2 (33.3) / 2 (33.3)	2 (10.0) / 2 (10.0)	2 (15.4) / 2 (15.4)	0.381 / 0.381
Craniotomy	n, (%)	3 (7.6) / 3 (7.6)	1 (16.7) / 1 (16.7)	1 (5.0) / 1 (5.0)	1 (7.7) / 1 (7.7)	0.643 / 0.643
Laparotomy	n, (%)	1 (2.5) / 1 (2.5)	0 (0.0) / 0 (0.0)	1 (5.0) / 1 (5.0)	0 (0.0) / 0 (0.0)	0.614 / 0.614
ORIF	n, (%)	0 (0.0) / 14 (35.9)	0 (0.0) / 3 (50.0)	1 (5.0) / 6 (30.0)	0 (0.0) / 5 (38.5)	0.614 / 0.651
Outcome						
DVT (+)	n, (%)	6 (15.4)	1 (16.7)	4 (20.0)	1 (7.7)	0.629
In-hospital mortality	n, (%)	2 (5.1)	0 (0.0)	1 (5.0)	1 (7.7)	0.779

Values are presented as median (interquartile range: IQR) or number (%), if appropriate

*Measurements of 38 patients. We started to measure IL-6 from the second patient of this study

The parameters reflecting the systemic inflammatory response and coagulofibrinolytic activation notably increased on day 0 (IL-6, 108.5 [40.8–250.3] pg/mL; TAT, 88.0 [30.1–200.0] μg/L; PIC, 9.1 [2.8–17.8] μg/mL; FDP, 63.0 [31.4–168.1] μg/mL). Eleven patients met the JAAM DIC criteria and 17 patients required transfusion. Two patients (5.1%) died from brain swelling due to severe head injury after the 7-day study period.

### ADAMTS13 activity on day 0 and its changes during the study period (Table 1 and Fig. 1)

**Fig. 1:**
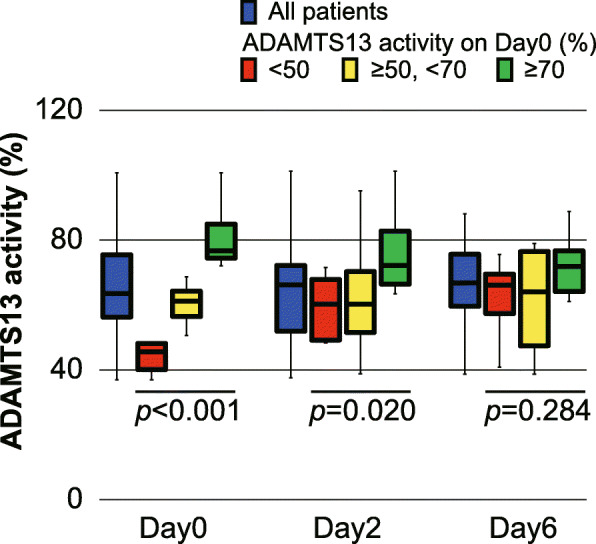
Time course changes in ADAMTS13 activity. Time course changes in ADAMTS13 activity of all patients are shown in blue columns. The central horizontal bars, columns, and peripheral longitudinal bars indicate the median values, 25th to 75th percentiles and 10th to 90th percentiles, respectively. The patients were stratified into three groups according to ADAMTS13 activities on day 0 (< 50%, red columns; ≥50 and < 70%, yellow columns; ≥70%, green columns). The differences in ADAMTS13 activities on day 0 among the three groups gradually decreased during the study period

The median ADAMTS13 activity of all the patients on day 0 was 63.6 (56.4–74.5)%. Six of 39 patients (15.4%) showed moderately decreased ADAMTS13 activity (< 50%), and 20 patients (51.3%) showed a slight depletion (≥50 and < 70%). The median values of the moderately decreased group, the slightly decreased group, and the normal group were 44.6 (42.1–47.7)%, 61.2 (56.5–63.0)%, and 76.7 (75.5–82.5)%, respectively (*p* < 0.001) (Table 1). However, the differences in ADAMTS13 activities among the three groups disappeared following the study period (*p* = 0.84) (Fig. 1).

### Correlation between ADAMTS13 activities and the coagulofibrinolysis related parameters on day 0 (Figs. 2 and 3 and Tables 1 and 2)

**Fig. 2:**
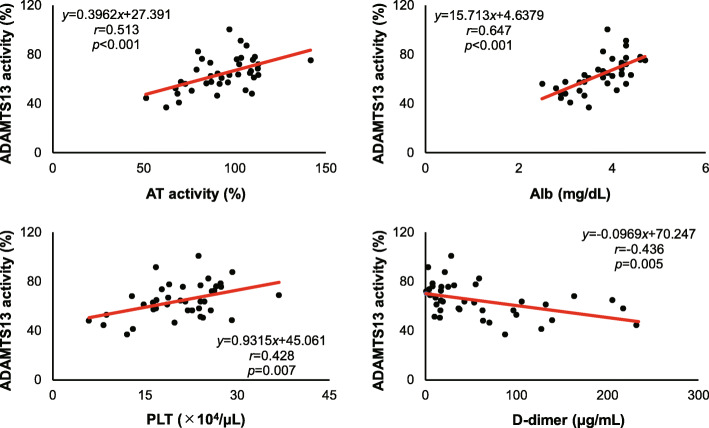
Single linear regression analyses of ADAMTS13 activities with AT activities (upper left), serum Alb levels (upper right), platelet counts (lower right), and D-dimer levels (lower left) on day 0. *r*, correlation coefficient; *p*, *p* value

ADAMTS13 activities on day 0 were significantly correlated with IL-6 values at the same point (*p* = 0.037), but not with ISS or Lactate (*p* = 0.75, *p* = 0.43) (Table 1). The coagulofibrinolytic markers including PLT, PT, HPT, FDP, D-dimer, PIC, AT, PC, α_2_PI and Alb were also correlated with ADAMTS13 activities on day 0 (*p* < 0.05) (Table 1, Fig.2). Though insignificant, there was also a trend of correlations between ADAMTS13 activities and the values of other coagulofibrinolytic parameters (Table 1). Serum Alb levels and plasma AT activities showed significant linear correlations with ADAMTS13 activities (Alb: *r* = 0.647, *p* < 0.001; AT: *r* = 0.513, *p* < 0.001), which were accompanied by significant correlations of platelet counts and D-dimer levels with ADAMTS13 activities (PLT: *r* = 0.428, *p* = 0.007; D-dimer: *r* = 0.436, *p* = 0.005) (Fig. 2).

**Fig. 3:**
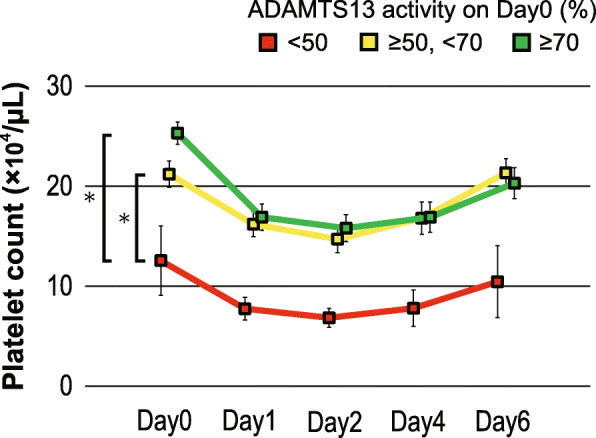
Time course changes in platelet counts. The central boxes and peripheral horizontal bars indicate the median values and 25th to 75th percentiles, respectively. The time courses of platelet counts are significantly lower in patients whose ADAMTS13 activities were less than 50% on day 0 than in those whose ADAMTS13 activities were at least 50% on day 0. * *p* < 0.05

**Table 2:** Simple logistic analyses for outcomes and treatments associated with the decreased ADAMTS13 activity to less than 50% on day0 (*n* = 39)

	*OR* (95% *CI*)	*P value*
Development of DVT	1.120 (0.107, 11.726)	0.925
Development of DIC	7.429 (1.121, 49.244)	0.038
Requirement of transfusion of FFP	9.000 (1.327, 61.025)	0.028

*OR* odds ratio, *CI* confidence interval

The moderately decreased ADAMTS13 activities to less than 50% on day 0 were significantly associated with the consumption of platelets (Fig. 3). Simple logistic regression analyses showed that ADAMTS13 activity when decreased to the levels less than 50% was significantly correlated with the development of DIC (*OR* 7.429, 95%*CI* 1.121–49.244, *p* = 0.038) and the need for transfusion of fresh frozen plasma (FFP) (*OR* 9.000, 95%*CI* 1.327–61.025, *p* = 0.028) (Table 2). However, the decreased ADAMTS13 activity was not correlated with the development of DVT (*OR* 1.120, 95% *CI* 0.107–11.726, *p* = 0.925) (Table 2).

## Discussion

### Plasma levels of ADAMTS13 activity in trauma patients

Suppressed ADAMTS13 activity less than 10% [4, 7, 8] is a cause of TTP, showing remarkable platelet consumption leading to thrombotic microangiopathy (TMA). Alternatively, there are slightly or moderately decreased ADAMTS13 activities with secondary thrombotic disorders such as sepsis [16–20] or other conditions [21–23]. However, ADAMTS13 has not been evaluated in adult trauma patients. Recently, in pediatric trauma patients, Russel et al. reported slightly decreased ADAMTS13 activities from 74.3 to 83.0% during the first 24 h [10]. In the current study of adult trauma patients, the median ADAMTS13 activity on day 0, which means the condition of just starting the initial treatments in the emergency room as shown in the method section, was 63.6%. Approximately 66.7% of the patients showed decreased ADAMTS13 activity below the lower limit of the normal range of 70%. Furthermore, a reduction to less than 50% was observed in 15.4% of all the patients. These results indicate that ADAMTS13 activity could decrease even in the early phase of adult trauma patients.

### Mechanisms of decreased ADAMTS13 activity in trauma patients

Secondary deficiency of ADAMTS13 are reported due to a reduced production in the liver [24], consumption through ongoing cleavage of ULvWF [2, 25], inhibition by inflammatory cytokines [26], cleavage by neutrophil elastase [27], plasmin, or thrombin [27, 28]. The present results showed that the decreased ADAMTS13 activity in the very early phase of trauma was correlated with the changes in coagulofibrinolytic markers and serum levels of IL-6, so that the possible mechanisms in the previous reports describing as coagulopathy and systemic inflammation could be supported by the present results. As shown in the Fig. 2, another major finding of this study is that ADAMTS13 activities showed positive correlations with AT activity and serum Alb levels even on day 0. AT and Alb decrease in the acute phase of the critical conditions such as sepsis [29–31] or trauma [32] possibly through vascular leakage from its augmented permeability [33, 34]. So, also in trauma, there is a possibility that vascular leakage might be involved in the changes in AT and Alb levels with ADAMTS13 activities.

On the other hand, we showed also in the Fig. 2 that the correlations between platelet counts and D-dimer levels with ADAMTS13 activities were significant, so, these correlations might imply thrombus formation due to trauma induced hypercoagulation. However, all these correlations depicted in the Fig. 2 were not so robust, which suggest that some other factors also might affect ADAMTS13 activity in the very early phase of trauma. So, we need to define such points in the future.

### Clinical significance of decreased ADAMTS13 activity in trauma patients

In our current adult trauma study, moderately decreased ADAMTS13 activities (< 50%) on day 0 were significantly associated with platelet depletion, development of DIC and a need for transfusion of FFP. Furthermore, the decreased ADAMTS13 activities on day 0 were improved over time, which was most likely due to transfusion of FFP for the treatment of coagulopathy after trauma.

Ono et al. reported that in septic DIC patients, a severe reduction in ADAMTS13 activity developed and suggested that sepsis may have a similar condition of severe ADAMTS13 deficiency for developing TMA. They also implied a potential supportive therapy of ADAMTS13 supplementation in sepsis [20]. Trauma also activates coagulofibrinolytic responses to occasionally provoke trauma-induced coagulopathy (TIC), which could eventually cause DIC. TIC with systemic inflammation and endothelial cell injury may cause ADAMTS13 decrease [10], which might be a risk of further platelet consumption and thrombus formation [35]. Transfusion of FFP could supply not only coagulation factors but also ADAMTS13, which might result in suppressing excessive platelet aggregation and exhaustion, or stabilizing endothelial conditions, possibly by increasing ADAMTS13 levels [36].

The levels of decrease in ADAMTS13 seem to play important roles in the disease severity and thrombotic disorders. However, cut-off values of ADAMTS13 activity that could predict the prognosis and the development of thrombotic disorders vary across different critical conditions. In the current study, we showed the actual influence of decreased ADAMTS13 activity on coagulation activity or thrombus formation. So, in the future, we need to clarify the range of ADAMTS13 activity differentiating the pathophysiological severity, which could contribute to determine treatment interventions.

### Limitations of this study

Several limitations of the present study should be addressed. First, the sample size of this study was small, so even though we obtained the present results by the appropriate statistical methods, we need larger-scale studies to clarify our hypotheses. Second, we did not assess the levels of vWF and its multimer which directly affects coagulation associated with ADAMTS13 in this study, so we need to examine them to clarify the pathogenesis of the association between ADAMTS13 and trauma-induced coagulopathy. Third, we included cases with mild trauma severity (i.e., those who survived for longer than 7 days), but one of the aims of this study was to examine the serial time course changes in ADAMTS13 activity in adult trauma patients, which have not yet been examined. We therefore might need to include such patients with mild severity. Even after the inclusion of such patients, we detected the clear coagulofibrinolytic responses and ADAMTS13 reductions. However, further studies, including more severe cases, might be required to determine the hemostatic status under different conditions. Fourth, we did not assess a sufficient range of issues regarding the site of trauma, since many patients included in this study suffered from blunt trauma to multiple organs. Therefore, there is the possibility that coagulofibrinolytic responses differ depending on the injured organs especially if the brain is involved. Accordingly, a future study considering the injured sites of trauma is needed to clarify this point.

## Conclusions

ADAMTS13 activity decreased even in the early phase of trauma, which was correlated with inflammatory and coagulofibrinolytic responses. Furthermore, the decreased ADAMTS13 activity was correlated with the development of DIC and the requirement of transfusion of FFP. Severe trauma patients with ADAMTS13 activity reductions might experience complications from coagulopathy.

## Data Availability

The datasets for the current study are available from the corresponding author upon reasonable request.

## Abbreviations

ADAMTS13: The 13th member of a disintegrin-like and metalloprotease with thrombospondin type 1 motif
AT: Antithrombin
TAT: Thrombin-antithrombin complex
Alb: Albumin
sTM: Soluble thrombomodulin
t-PA: Tissue plasminogen activator
PC: Protein C
tPAI-1: Total plasminogen activator inhibitor-1
PT: Prothrombin time
APTT: Activated partial thromboplastin time
Fbg: Fibrinogen
FDP: Fibrin/fibrinogen degradation product
PIC: Plasmin-α_2_-plasmin inhibitor complex
α_2_PI: α_2_-plasmin inhibitor
PLG: Plasminogen
TIC: Trauma-induced coagulopathy
DIC: Disseminated intravascular coagulation
ISS: Injury Severity Score
JAAM: The Japanese Association for Acute Medicine
IVR: Interventional radiology
ORIF: Open reduction and internal fixation
